# Increasing Registered Nurse Hours Per Resident Day for Improved Nursing Home Residents’ Outcomes Using a Longitudinal Study

**DOI:** 10.3390/ijerph18020402

**Published:** 2021-01-06

**Authors:** Juh Hyun Shin, Rosemary Anne Renaut, Mark Reiser, Ji Yeon Lee, Ty Yi Tang

**Affiliations:** 1 College of Nursing, Ewha Womans University, Seoul 03760, Korea; dlwldusking@hanmail.net; 2 School of Mathematical and Statistical Sciences, Arizona State University, Tempe, AZ 85281, USA; renaut@asu.edu (R.A.R.); mark.reiser@asu.edu (M.R.); 3 Department of Psychology, Arizona State University, Tempe, AZ 85281, USA; tytang24@asu.edu

**Keywords:** NHs, workforce, nurses

## Abstract

The purpose of this study was to estimate how much resident outcomes can improve with an increase in hours per resident day (HPRD) of registered nurses (RNs) staffing. Nursing home (NH) staff in Korea have serious problems with inappropriate nurse staffing standards and poor working conditions, which lead to poor quality of care for NH residents. This study used a longitudinal survey design. A quota sampling was used with a total of several repeated survey measurement from 2017 to 2020 (*n* = 74). The independent variable was the amount of nurse staffing HPRD and the outcome variable was the compiled outcome of 15 quality-of-care indicators. Data were directly collected from all participating NHs. A longitudinal, multilevel model was used for analysis. An increase of one unit of RN HPRD (60 min) corresponded to a decrease of about 10.5% of residents with deteriorated quality of care outcomes. This study emphasized that increasing RN HPRD decreased residents’ deteriorated outcomes in NHs. This suggests that professional RNs must be secured to an appropriate level to improve the quality of care for NH residents.

## 1. Introduction

Researchers of nursing home (NH) staffing during the last decades have reported the enormous importance of appropriate number of registered nurses (RNs) on staff in residents’ outcomes worldwide, yielding diminished numbers of pressure ulcers, falls, use of restraints, pain, and reduced decline in the activities of daily living (ADLs) [1,2,3,4,5,6,7,8]. Along with the number of RNs, the major role of RNs in NHs is very important for care, including assessment of physical or psychological symptoms. RNs develop scientific care plans based on the timely assessment of residents’ health status and cooperate with physicians for better outcomes [9]. RNs in NHs supervise other nursing staff including licensed practical nurses (LPNs) or certified nursing assistants (CNAs). Also, RNs share responsible for scheduling the nursing staff [9,10]. To determine if there is an insufficient number of RNs in NHs, a suitable assessment based on evidence is not possible, resulting in “failure to rescue.” [11].

NHs have a larger portion of RNs reported as having a higher public reporting grade [12,13]. Despite the lack of difference between hospital patients’ and residents’ outcomes in the difficulty of caring for their needs, historically, NHs have lacked RNs. Due to the apparent significant positive role of RNs in long-term care in resident outcomes, public reporting continuously calls for mandatory staffing requirements [9]. Previous research has provided evidence of legislatively mandated nurse staffing in NHs. For example, the Omnibus Budget Reconciliation Act of 1987 required a minimum of 0.08 h per resident day (HPRD; hours worked by RNs divided by the total number of residents) [14] for RNs since 1987 in the Unites States [15]. On 12 February 2015, a U.S. law required at least one registered professional nurse be assigned in NHs to provide assessment, surveillance, and direct care to residents, 24 h a day, seven days a week (H.R. 952) [16].

The major nurse-staffing issue in Korea is that NHs do not have sufficient numbers of RNs. For example, 79% of NHs do not have any RNs, although they adhere to the legal requirements. The legal criteria for staffing is that each NH has one RN or CNA per 25 residents [17]. Nurse staffing in Korean NHs includes RNs, CNAs, and qualified care workers. Korean NH CNAs are the workforce who help RNs in emergency situations, providing basic wound treatment, bathing, feeding, and transporting residents [17]. Most RNs in Korea try to work in acute settings and metropolitan areas [18] because of low wages and poor working conditions in NHs compared with acute settings. For these reasons, the supply of RNs in the Korean NH industry is insufficient. The number of NHs is 3389; the number of residents is 163,484; and the number of RNs is 1472 [19]. The average number of RNs per NH was reported as 0.43 [19]. Also, data on HPRD in Korean NHs was unavailable because the government collects only the number of each staff. A few studies collected data on HPRD and reported that RNs’ HPRD in NHs was 6 min 29 s–11 min 5 s [20]. Korean NHs’ HPRD is quite insufficient compared to the United States [21].

There have been, however, no systematic reviews that investigate a minimum HPRD of RNs to attain the maximum quality of care in NHs [22]. Most studies used cross-sectional or retrospective designs [22]. In a systematic review (54 studies in total), 20 (37%) were retrospective studies, 16 (30%) were retrospective studies with cross-sectional analysis, and nine (17%) used cross-sectional analysis. Of these, only two used a longitudinal design [23]. Conclusions derived from such designs may be biased if there are unobserved time-invariant factors that affect NH quality or resident health status [23]. These factors may correlate with the explanatory variables of the study. Due to the limitations of the study design, studies of the relationship between nurse staffing and quality of care in NHs have shown inconsistent results [22].

Major concerns raised through past research were that the effect of RNs on outcomes may be impacted by regulations, residents’ case mix, and financial resources. Also, researchers may take factors too lightly, failing to control for residents’ case mix [9,24]. However, this study used a longitudinal dataset to overcome possible biases that may explain time-dependent undetected heterogeneity [9]. All health conditions other than the variables in this study that change over time could not be controlled. This study’s longitudinal design mitigates that problem. Also, previous studies did not consider covariate variables on outcomes without considering organizational and market factors [25]. This study considered organizational and market factors simultaneously such as location, ownership, size of NH, facility evaluation by the Korean National Insurance Corporation, and HHI.

The preceding studies are as follows. Only one previous study estimated the increase of HPRD on quality-of-care improvement using an optimization model [5]. The optimization model is an algorithm that scientifically seeks values of variables to make the most effective decision in the context of constraints [26]. In the Shin [5] study, a 12% increase in RN HPRD (from 0.168 HPRD (10 min 5 s) to 0.177 (10 min 38 s)) aligned with a 3% improvement in quality-of-care outcomes. A 20% RN HPRD increase aligned with a commensurate 5% to 8% increase in compiled quality-of-care outcomes (from 0.168 HPRD (10 min 5 s) to 0.202 HPRD (12 min 6 s) [5]. The objective of this study was to estimate how much resident outcomes can improve with an increase in HPRD of RN staffing. The hypothesis was set as follows: “Higher RN HPRD relates to lower deteriorated quality of resident-care outcomes in NHs” and “Higher RN HPRD in total nurse staffing HPRD relates to lower deteriorated quality of resident-care outcomes in NHs”.

## 2. Methods

### 2.1. Study Design

A secondary analysis of a longitudinal survey of NH data in Korea was used. Data were retrieved from a 3-year research project that was conducted between February 2017 to February 2020. An institutional review bard of a university in Korea approved the parent study (No. 136-4).

### 2.2. Sampling

The quota sampling method was applied according to an area distribution of NHs across Korea. A NH list was used (a total NHs *n* = 1647, with more than 29 beds) provided by the Korean National Insurance Corporation. Disproportionate stratified random sampling was used to gain geographical representation across Korea and divided Korea into subgroups aligned with the 17 administrative districts; NH distribution ranged from about 0.3 to 32.5%, by administrative district. One to 20 NHs were randomly selected in each district (a value equal to 10% of the number of NHs distributed in each district) and a total of 170 NHs were contacted. Administrators of 74 NHs agreed to participate and signed consent forms (response rate: 43.53%). The response rates were 100% (1st), 86% (2nd), 90% (3rd), 92% (4th), 85% (5th), 83% (6th), and 77% (7th), with an overall average response rate of 88%.

To minimize the attrition rate, financial incentives of $100 per survey completion were provided and continuous participation was encouraged through continuous emails and visits to NHs. The attrition of data collection occurred as a result of NH businesses closing, resignations of administrators, and refusal due to staff shortages, or due to survey responses required during the accreditation period.

### 2.3. Data Collection

Prior to data collection, the principal investigator of this study provided education and training to the three research assistants on the purpose and procedures of this study, how to obtain consent forms, and how to collect and code data. The three research assistants collected data, one of whom obtained the list of NHs, one coded the data, and one handled monetary incentives for NHs participating in this study. After completion of coding by the first research assistant, the second assistant checked the codes to increase data accuracy.

Data included a total of seven repeated survey measurements (one every 3 months) through e-mail, phone, or physical visits on nurse staffing HPRD and 15 quality indicators. NHs in Korea are not equipped with a minimum data set, Online Survey Certification and Reporting System, epic system, Survey on patient Safety Culture NH database, or Skilled Nursing Facility data, which are used for clinical assessment data of residents in NHs. Because specific information on staffing information and the characteristics of residents (i.e., sex, age, health outcomes, and so on) were unavailable for those sources, that information was collected directly from participating NHs.

The 15 quality-of-care indicators from the 88 quality-of-care indicators in the NH evaluation manual published by the Korean National Health Insurance Corporation were selected [27]. Nursing-sensitive outcome indicators were cognitive impairment, urinary incontinence, antidepressant of sleeping pill, fecal incontinence, bed rest, physically restrained, tube feeding, aggressive behavior, depression, fall prevalence, help for daily living, slip prevalence, hospital admission, range of motion, 5% weight loss and 10% weight loss, pressure sore prevalence, and dehydration. Another 73 indicators were not related to nursing-sensitive outcomes including physical environment or adherence with law. The formula to calculate the 15 quality indicators followed the U.S. Minimum Data Set calculation method [28].

HPRD is the average hours worked by each type of staff (RNs and CNAs), divided by the total number of residents [28], in this case using only nursing-sensitive indicators.

In addition, number of RNs was collected in each administrative district from the open-access Korean National Health Insurance Corporation Herfindahl–Hirschman Index (HHI) to measure health-care market competition [29] and concentration of NHs in the health care industry through HHI. The number of RNs in each participating NH was divided by the total number of NHs in each of the 17 administrative districts. Then, the result squared for each of the participating NHs. NHs were grouped depending on the administrative districts and the total HHI index summed of all NHs in the 17 administrative districts. Higher values mean more competition (range: 0–1). The long-term care insurance hierarchy (five stages reflecting residents’ case mix in Korea), rests on the degree of care and services provided to residents [29].

### 2.4. Covariates

In this study, institutional factors, including location, ownership characteristics, size of the facility, facility evaluation by Korean National Insurance Corporation, and HHI served as control variables.

### 2.5. Analysis

R statistical programming language was used to perform the analysis [30]. A longitudinal multilevel model was used, as follows: for all models, individual resident outcome measures are divided by the number of residents. Aggregate *z*-scores were calculated as aggregated sums of *z*-scores for all individual-resident outcome measures. For reducing the size of the problem and calculating time with numbers, outcome variables (15 quality indicators) were summed [31]. Outcomes reflect the aggregated quality of care outcomes. The multilevel model used only one intercept and categorical institution for the second level, so there was no “change in outcomes” associated with the second level of the model.

RN_HPRD on Outcomes

$$Yijkl\;=\;\beta_{0}\;+\;\beta_{1}\mathrm{RN}\_\mathrm{HPRD}jkl\;+\;\beta_{2}\mathrm{SkillMix}jkl\;+\;Xjkl\;+\;\alpha_{i}\;+\;\alpha_{l}\;+\;\varepsilon ijkl$$

where *i =* NH, *j* = market factor(HHI), *k* = province, *l* = time, *α_i_* = size of NH (fixed effect), *α_j_* = operation (fixed effect), *α_k_* = Residents’ case mix, and *α_l_* = location.

## 3. Results

Table 1 provides the descriptive characteristics of the 74 participating NHs, staff, and residents. At the organizational level, the prevalence of cognitive impairment was 67.76%, urinary incontinence was 42.10%, use of antidepressant sleeping pills was 27.38%, fecal incontinence was 22.62%, bed rest was 21.91%, physical restraint was 6.40%, tube feeding was 6.36%, aggressive behavior was 5.62%, depression was 5.45%, fall prevalence was 4.63%, help for daily living was 4.24%, slip prevalence was 3.46%, hospital admission was 2.67%, range of motion was 2.51%, 10% weight loss was 1.69%, 5% weight loss was 1.35%, pressure-sore prevalence was 1.27%, and dehydration was 0.72%. In terms of organizational characteristics, 81.0% of NHs participating in this study were nonprofit, whereas only 3.1% of NHs in Korea are nonprofit [32]. The average number of beds per NH participating in this study was 72.06; this is higher than the average number of beds (33.25) for all NHs in Korea [33]. About 36.49% of the participating NHs received a superior grade in facility evaluation by Korean National Insurance. In contrast, only 13.4% of all NHs in Korea received a superior grade and about 21.1% of NHs received the lowest grade in the most recent facility evaluation [33]. The average RN HPRD was 0.179 (10 min 45.1 s), CNA HPRD was 0.335 (20 min 6.8 s), and care worker HPRD was 3.545 (3 h 32 min 43.4 s) in this study. In very limited previous studies, the average RN HPRD was 0.1–0.2. about (6 min 48 s–11 min 5 s), CNA HPRD was 0.2 (about 9–10 min 31 s) [4,6,7]. The average number of RNs per NH was 1.567, the minimum was 0, and the maximum was 17. Participating NHs in this study had a higher ratio of RN to residents (1:42) than general NHs in Korea (1:106) whereas the ratio of CNAs and care workers to residents were quite similar to the Korean national averages (CNAs: 1:35 study, 1:21 nationally; care workers: 1:2 study, 1:2 nationally) [33].

Also, Table 2 provides the change of RN HPRD and quality of care according to each survey point (7 times).

In residents’ quality-of-care outcomes, RN HPRD was the only significant variable. An increase of 1 unit of RN HPRD (equivalent to 60 min) corresponded to a decrease of about 10.5% of residents with deteriorated quality of care outcomes. In this study, a multilevel longitudinal model was estimated and established using restricted maximum likelihood, which is not sensitive to outliers or missing values of data as compared to maximum likelihood [34]. The final model fit results are estimated by the restricted maximum likelihood (see Table 3). Among the various covariance matrices, the Akaike information criterion and Bayesian information criterion are the best ways to find the most suitable matrix. These values impose a penalty on the number of parameters on the value of log likelihood the lower the value, the more appropriate it is for the model [35].

## 4. Discussion

Previous studies provided limitedly reports of the relationship between nurse staffing and resident outcomes. A higher HPRD of RNs providing care aligned with fewer pressure ulcers, [10,36,37] fewer falls, [38] less cognitive impairment, [7] fewer urinary-tract infections, [19,35] less tube feeding, [4,29] decreased weight loss [39], decreased deteriorated range of motion [38], decreased numbers of residents with depression [4], decreased residents with psychotropic medications [7], decreased rehospitalizations and emergency-department visits [3], reduced restraint use [10], more out-of-bed activity [4], more exercise and repositioning [38], more improved functional ability [39], and increased quality-of-care outcomes [6].

This study analyzed the three-year longitudinal NH datasets of RN HPRD and quality-of-care outcomes. Study results showed a statistically significant 10.5% improvement in residents’ sum of the 15 quality of care indicators when increasing one unit of RN HPRD (60 min). This study contributed to the literature in that outcomes suggested an amount of change in outcomes. Although very few studies have been conducted, results supported a higher level of RNs and quality-of-care services [4,5,7,38,40]. In a study in the United States, increasing the number of RNs by 0.3 HPRD increased quality of care by more than 16% [9]. This figure is equivalent to lowering the number of deficiencies from 7.4 to 6.2 [9]. In another study, the required RN HPRD was reported as 0.31, 1.8, and 3.3 to reach quality improvements of 50%, 75%, and 90% [41]. Consistent with the very limited previous research about the increase of RN HPRD and outcomes, the increase of RN hours for residents was effective for residents outcomes, indicating a 3–8% increase in the quality of residents’ outcomes with a very small increase of 12–20% input from RNs (from 10 min 5 s to 10 min 38 s and from 10 min 5 s to 12 min 2 s). The addition of 60 min of RN HPRD yielded 10% better quality-of-care outcomes. Despite collecting data on all 15 variables separately, each outcome’s relation to HPRD could not be examined because of the small sample size. Instead, outcomes were compiled to decrease the risk of inappropriate calculations [42].

RNs are generally key human resources in residents’ outcomes. Specifically, RNs assess and remove fall-risk factors of residents in NHs and create NH environments that prevent falls. Thus, an increase in RN’s HPRD affects the fall prevalence of residents [38]. RNs communicate with doctors authorized to prescribe antipsychotic medications to prevent overuse of antipsychotics and plan and provide nonpharmaceutical interventions, thereby influencing the rate of use of antipsychotic drugs [7]. RNs play an important role in assessing the nutritional status of the residents, such as poor appetite, lack of food intake due to disease, and interactions with food and drugs, and provide systematic nutrition programs for NH residents. This role of RNs has a positive impact on preventing residents from losing weight [39]. Also, RNs assess difficult parts of daily life such as washing, combing hair, dining, and walking of residents, motivating them to carry out their activities of daily living independently and encourage step-by-step strategies, as well as educate and supervise nursing assistants to perform these roles. This skill leads to an increase in the ability of elders to perform their activities of daily living [4,39]. As the time of RN’s HPRD in NH increases, they enhance the range of motion related to the timely assessment of physical changes of residents and secure more time for residents to take part in muscular exercises [38].

South Korea introduced public long-term-care insurance (Korean legal system for the elderly) in July 2008 [33]. As of 2020, 3390 NHs operated in 17 geographical areas in Korea. Private owners operate most NHs (60.9%) in Korea, followed by corporations (36%) and by local governments (3%) [32]. The required total nursing HPRD by law [40] is between 0.32 (19 min 2 s) to 0.9 (54 min), including both RNs and CNAs. In this study, The RN HPRD of this study was 0.179 (10 min 45.1 s), the CNA HPRD was 0.335 (20 min 6.8 s). The time-varying RN HPRD showed that RN HPRD is three and four times higher over seven survey periods across three years. However, there was not much change in the number of NH residents, but the number of RNs temporarily increased at that time. This could also be linked to the introduction of an additional payment system for NHs for the deployment of RNs at NHs in Korea [17]. The Korean government implemented a RN placement or additional pay system in which an NH deploys and operates more than one RN according to the number of RNs [17]. However, the low level of additional pay points does not fully reflect the role of RNs and the level of labor costs, which does not encourage hiring and deploying RNs from a long-term perspective.

HPRD by RNs and CNAs was differentiated, showing that RN HPRD was very low compared with the median RN HPRD in the United States (4 h 25 min) [43]. The reason HPRD of NH RNs in Korea is significantly lower than that of the United States is that U.S. law requires at least one RN be assigned [16], whereas the Korean nursing staff regulation is a one RN or CNA per 25 residents. In other words, because CNAs are allowed to replace RNs, most NH administrators hire CNAs to reduce costs. Also, RNs are reluctant to work in NHs due to low wages and poor working condition [44]. As results of this study and prior research, because fewer RNs’ HPRD relates to deterioration of quality-of-care outcomes, it is important that regulatory standards for the placement of nursing staff in NHs in Korea should be stipulated to mandate NHs have RNs on staff. In addition, government support is needed to improve salaries and working conditions of RNs in NHs and improve the work environment, encouraging more RNs to work in NHs. Furthermore, the use of gerontology nurse practitioners (GNP) is recommended. GNPs have completed a nurse-practitioner-specific master’s program. GNPs independently assess patients, perform physical examinations, order diagnostic tests, interpret diagnostic tests, diagnose, make appropriate referrals, and prescribe medications in their collaborative scope of practice [45]. Considering residents’ improved outcomes such as length of stay, quality of life, satisfaction, and cost are reported when GNPs are deployed in NHs, the role of GNPs is important [46]. South Korea needs to initiate having nurse practitioners as well as primary care physician in NHs. Korea had 2174 GNPs as of 2017. However, they could not practice as nurse practitioners because very limited infrastructure is available to hire nurse practitioners [47].

Meanwhile, in this study, a random sampling method was used, but the various characteristics of the NH are quite different from those of Korean NHs. A total of 170 NHs were contacted, with 74 NHs participating. This study considered both types of NHs where RNs are deployed and those where they are not. Since the objective of this study was to report the importance of RNs in NHs, it was more favorable for participants to come from those institutions employing RNs. Also, there are two types of NHs (for-profit and non-profit) in Korea, and because for-profit NHs are for profit purposes, CNAs are hired instead of RNs due to financial issues. Therefore, non-profit NHs hire more RNs. Considering these facts, it is inferred that this study is the reason why NHs with more RNs were recruited than the Korean average and more non-profit NHs were selected. In relation to bed size, the smaller bed size the distribution of Korean NHs, the more profitable they are. Since many non-profit NHs participated in this study, the number of beds is also interpreted as high.

Most Organization for Economic Co-operation and Development (OECD) countries, including South Korea, reported that more RNs in hospitals yielded higher quality patient outcomes [39]. Compared with OECD countries, Korea ranks very low (fourth from bottom) in ratio of RNs to population; 18 to 1000 people in Switzerland, 17:30 for Norway versus 5.9:1000 in Korea [48]. It is imperative to improve long-term care service for residents in NHs by presenting scientific input from nursing staff on outcomes. The results of this study confirmed that the increase in the HPRD of RNs in NHs related to improvement in quality-of-care outcomes, thereby becoming an important basis for the need for increased HPRD of RNs in the enactment of nursing-staff regulations worldwide. Results also help determine the optimal staffing to improve quality-of-care outcomes. In long-term care settings, U.S. federal law (H.R. 952) [16], which requires mandatory RNs in NHs, is consistently supported by the American Nurses Association, Coalition of Geriatric Nurses Organization, National Consumer Voice, and American Geriatric Society [11].

## 5. Conclusions

This study reported that an increase of one unit of RN HPRD (60 min) corresponded to a decrease of about 10.5% of residents with deteriorated quality of care outcomes. In other words, this study provides evidence that increasing RN HPRD improved residents’ quality of care outcomes in NHs. This is because professional RNs can develop scientific care plans based on the timely assessment of residents’ health status and cooperate with physicians for better outcomes. This suggests that professional RNs must be secured to an appropriate level to improve the quality of care for NH residents.

The accumulation of nurse-staffing research on residents’ outcomes continuously supports more RN hours. The long-term-care law in Korea that regulates NHs, however, does not yet apply these research findings. This study again supports the regulation adjustment of nurse staffing and legislation, necessitating professional RNs to accomplish appropriate quality of care for residents in Korean NHs.

### Limitation

This study had some limitations. First, participating NHs may not reflect Korean NHs as a whole, which could bias the estimates. Although about 36.49% of participating NHs in this study received a superior grade, only 13.5% of total NHs in Korea had a superior grade on the 2018 NH evaluation by the Korean National Health Insurance Corporation [33]. Also, about 60% of NHs are for-profit organizations [32], whereas 18% of NHs in this study were for-profit organizations. Furthermore, the ratio of RNs to residents in this study (1:3) is higher than the average ratio in general in Korea NHs due to NH employing RNs would have been more cooperative [33]. Thus, external validity may be threatened. Thus, more research should be conducted to examine outcomes with larger samples to reflect all Korean NHs. However, it was tried to examine the representativeness of NHs that participated compared to NHs in Korea overall by comparing nonparticipating NHs’ organizational information. In addition, this study focused on the structure of nurse staffing and could not measure the actual process of nurse staffing including direct care or indirect care, both simultaneously impacted by regulating direct care; NH care is clearly impacted by nursing staff directly [49,50]. Finally, further study should include other organizational covariates including working conditions, the psychosocial environment, social support, and neighborhood circumstances.

## Figures and Tables

**Table 1:** Organizational Characteristics of 74 NHs.

Demographics	Frequency (%)	Mean(SD)		Min–Max
Ownership				
For profit	14 (18.82)			
Not for profit	60(81.08)			
Operation duration (years)		11.27(5.35)		3–36
Bed size		72.06(52.53)		7–296
Occupancy rate		92.29(12.34)		40.00–103.45
Location of organizations				
Metropolitan (>1 million)	32(43.24)			
Medium size (500 thousand–1 million)	17(22.97)			
Small size (50–500 thousand)	18(24.32)			
Rural area (<50 thousand)	7(9.47)			
HHI		0.0006348 (0.0018310)		0.0000250–0.0324000
Facility evaluation by Korean National Insurance Corporation				
A grade ^a^	27(36.49)			
B grade ^b^	13(17.57)			
C grade ^c^	11(14.86)			
D grade ^d^	9(12.16)			
E grade ^e^	0(0.00)			
Excluded from evaluation ^f^	14(18.92)			
Hours Per Resident Day (Hour)				
RN		0.179(0.205)		0–1.030
CNA		0.335(0.206)		0–1.425
CW		3.545(1.350)		3.131–11.126
Director		0.243(0.165)		0–1.425
Secretary		0.132(0.129)		0–0.570
Social worker		0.407(0.293)		0–3.663
Administrative staff		0.139(0.169)		0–1.368
Dietician		0.091(0.089)		0–0.438
Cook		0.367(0.224)		0–1.425
Number of RN per facility		1.567(3.034)	0	0–17
Skill mix				
RN:CNA	1:1.20			
RN:CW	1:17.08			
Turnover rate				
RN		6.696(17.189)	0	0–100
CNA		14.077(23.710)	0	0–100
CW		16.187(15.807)	0	0–86.179
Quality of care (%)				
Cognitive impairment	67.76			
Urinary Incontinence	42.10			
Antidepressant of sleeping pill	27.83			
Fecal Incontinence	22.62			
Bed rest	21.91			
Physically restrained	6.40			
Tube feeding	6.36			
Aggressive behavior	5.62			
Depression	5.45			
Fall prevalence	4.63			
Help for daily living	4.24			
Slip prevalence	3.46			
Hospital admission	2.67			
Range of motion	2.51			
10% Weight loss	1.69			
5% Weight loss	1.35			
Pressure sore prevalence	1.27			
Dehydration	0.72			

Abbreviation. CNA = certified nurse aide; CW = care worker; HHI = Herfindahl–Hirschman Index: measure of market concentration; N/A = not applicable; RN = registered nurse, ^a^ Score of 90 or more, and 70 points or more of each major classification area. ^b^ Score of 80 or more, and 60 points or more of each major classification area. ^c^ Score of 70 or more, and 50 points or more of each major classification area. ^d^ Score of 60 or more, and 40 points or more of each major classification area. ^e^ Score of 59 or less, and 39 points or less in each major classification area. ^f^ Excluded from the evaluation because of administrative disposition due to the violation of long-term-care insurance laws.

**Table 2:** Change of RN HPRD and Quality of Care Outcome According to Each Survey Point (7 Times).

	1 Time	2 Times	3 Times	4 Times	5 Times	6 Times	7 Times
Hours Per Resident Day (Hour)							
RN	0.168	0.180	0.199	0.185	0.179	0.180	0.162
Quality of care (%)							
Cognitive impairment	70.83	67.88	65.58	66.53	64.51	63.58	75.41
Urinary Incontinence	41.11	42.76	42.25	41.12	40.20	44.98	42.28
Antidepressant of sleeping pill	28.88	27.34	25.56	28.87	26.60	28.12	29.44
Fecal Incontinence	21.67	29.18	26.78	26.21	26.21	27.12	27.38
Bed rest	21.89	22.32	20.91	19.98	20.11	23.56	24.60
Physically restrained	6.36	6.99	6.40	5.98	6.15	6.22	6.70
Tube feeding	6.75	6.34	6.48	6.12	5.99	6.23	6.61
Aggressive behavior	5.66	5.12	5.45	5.78	5.81	5.34	6.18
Depression	5.66	5.46	5.35	5.71	5.49	5.23	5.25
Fall prevalence	4.88	4.78	4.59	4.39	4.78	4.56	4.43
Help for daily living	4.24	4.34	4.28	4.29	4.21	4.54	3.78
Slip prevalence	3.45	3.46	3.26	3.49	3.26	3.86	3.44
Hospital admission	2.77	2.67	2.48	2.37	2.87	2.56	2.97
Range of motion	2.53	2.56	2.43	2.76	2.66	2.32	2.31
10% Weight loss	1.58	1.68	1.43	1.76	1.79	1.67	1.92
5% Weight loss	1.87	1.79	1.25	1.54	1.55	1.82	2.01
Pressure sore prevalence	1.34	1.32	1.15	1.24	1.35	1.22	1.27
Dehydration	0.78	0.71	0.67	0.69	0.78	0.65	0.76

**Table 3:** RN Hours per Resident Day and Quality of Care.

Outcome	Estimate	Standard Error	*df*	*t*-Value	*p*-Value	REML Criterion	Log Likelihood	Akaike Information Criterion	Bayesian Information Criterion
Aggregate *z*-	−10.59	4.37	187.07	−2.43	0.02	1380.41	−690.21	1392.41	1412.55

Abbreviation. REML = restricted maximum likelihood.
